# Nonlinear dose–response relationship between prognostic nutritional index and short-term outcome in acute ischemic stroke: a prospective cohort study

**DOI:** 10.3389/fnut.2025.1529146

**Published:** 2025-03-10

**Authors:** Juan Wang, Xiongbin Cao, Shan Zeng, Li Zhou, Jianping Huang, Yong Han, Zhe Deng

**Affiliations:** ^1^Department of Emergency, Shenzhen Yantian District People's Hospital, Southern University of Science and Technology Yantian Hospital, Shenzhen, China; ^2^Neurology Department, Shenzhen Longhua District Central Hospital, Shenzhen, China; ^3^Department of Emergency, Shenzhen Second People's Hospital, The First Affiliated Hospital of Shenzhen University, Shenzhen, China

**Keywords:** acute ischemic stroke, prognosis, non-linear relationships, prognostic nutritional index, modified Rankin scale score

## Abstract

**Objective:**

The evidence surrounding the connection between the Prognostic Nutritional Index (PNI) and the prognosis of patients with Acute Ischemic Stroke (AIS) remains insufficient. Therefore, this study is designed to examine how PNI relates to short-term outcomes in individuals affected by AIS.

**Methods:**

This study is a single-center, prospective cohort investigation. The study sample comprised 1,697 patients with AIS who received treatment at Shenzhen Second People’s Hospital between January 2022 and June 2024. To evaluate the association between the PNI and the risk of at 90-day unfavorable outcomes, as well as 90-day mortality, a binary logistic regression model was employed. Furthermore, a logistic regression model incorporating cubic spline functions was utilized to explore the potential non-linear relationship between PNI and 90-day unfavorable outcomes. Additionally, a series of sensitivity analyses and subgroup analyses were performed to enhance the robustness of the findings.

**Results:**

Following the adjustment for covariates, the binary logistic regression analysis demonstrated a notable inverse connection between PNI and the occurrence of unfavorable outcomes at 90 days among patients diagnosed with AIS (OR = 0.951, 95% CI: 0.925–0.979). A similarly significant negative relationship was found between PNI and 90-day mortality (OR = 0.868, 95% CI: 0.806–0.934). Additionally, the study revealed a non-linear association between PNI and 90-day, identifying an inflection point at PNI = 49.3. To the left of this inflection point, the OR for the risk of 90-day unfavorable outcomes in AIS patients was 0.910 (95% CI: 0.880–0.942). Conversely, to the right of the inflection point, the OR was 1.149 (95% CI: 0.998–1.249), although this finding was not statistically significant. The findings were further supported by sensitivity analyses, which reinforced the reliability of these results.

**Conclusion:**

This study reveals a significant negative association between the PNI and 90-day unfavorable outcomes as well as 90-day mortality in patients with AIS. A non-linear relationship between PNI and 90-day unfavorable outcomes was observed. Specifically, a significant inverse association between them was evident when PNI values were below 49.3. These findings offer valuable insights for refining rehabilitation strategies and improving the clinical management of AIS patients.

## Introduction

Acute ischemic stroke (AIS) is a prominent global contributor to disability and mortality, presenting a substantial socioeconomic challenge (1, 2). Although significant advancements have been made in both the acute management and rehabilitation of AIS, accurately forecasting neurological outcomes for affected individuals continues to pose a considerable challenge (3). Identifying prognostic indicators for patients with AIS is essential for effective risk stratification, the formulation of individualized treatment plans, and the enhancement of overall patient outcomes (4). Key prognostic factors that have been recognized in AIS include age, the existence of diabetes, the underlying cause of the stroke, and hypertension (5–7).

Malnutrition has been consistently associated with poor outcomes across various disease states, including heart failure, malignancies, and fractures (8–11). Among stroke survivors, malnutrition—stemming from complex interactions including comorbidities and dysphagia—is a prevalent condition, with its incidence varying dramatically from 3 to 87% (12). Previous investigations have demonstrated a significant correlation between nutritional status at hospital admission and subsequent clinical outcomes in stroke patients (13–15). The Prognostic Nutritional Index (PNI), a comprehensive metric derived from serum albumin concentration and peripheral lymphocyte count, serves as a robust indicator of both nutritional status and overall prognostic potential (16). Given the impact of malnutrition on the prognosis of patients with acute ischemic stroke (AIS), we hypothesize a potential relationship between the PNI and clinical outcomes in AIS patients.

Regrettably, the existing research on the relationship between PNI and unfavorable neurological outcomes in patients with AIS is quite limited. Most previous studies have focused on using predefined PNI cutoff points to categorize patients as malnourished or well-nourished and then explore the association between these groups and AIS-related outcomes. For instance, a study conducted in China involving AIS patients undergoing thrombolytic therapy reported that individuals with a low PNI (PNI ≤44.5) had a 1.25-fold higher incidence of poor prognosis within 3 months post-stroke, compared to those with a high PNI (adjusted OR = 2.250, 95% CI: 1.192–4.249) (17). Another study found that among patients with AIS, patients in the highest quartile of PNI had a lower risk of poor prognosis compared with the lowest quartile (OR = 0.40, 95% CI: 0.27–0.6) (18). However, few studies have investigated the relationship between PNI as a continuous variable and short-term outcomes in AIS or examined any potential nonlinear associations between them. Additionally, the existing research varies in terms of study design, PNI value ranges, gender distributions, adjustment factors, and definitions of functional outcomes. Furthermore, the relationship between PNI and short-term prognosis in AIS patients within the Chinese population remains unclear. Therefore, the present prospective cohort study aims to provide further insights into this specific relationship, which may inform rehabilitation strategies and help reduce the overall burden of AIS.

## Methods

### Study design and study population

This prospective cohort study was conducted at a single center, the Stroke Center of Shenzhen Second People’s Hospital in China, enrolling consecutive patients with Acute Ischemic Stroke (AIS) admitted between January 2022 and June 2024. The primary independent variable examined in this investigation was PNI measured upon hospital admission, while the outcome variables of interest were the 90-day functional prognosis and mortality among the AIS patient cohort.

**Figure 1:**
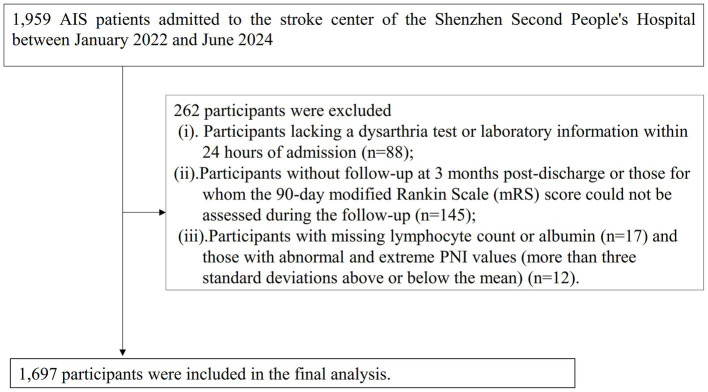
Flowchart of study participants.

This study enrolled patients diagnosed with ischemic stroke, confirmed by magnetic resonance imaging (MRI) or computed tomography (CT), who were consecutively admitted to the Stroke Center. The inclusion criteria were: (a) age ≥ 20 years; and (b) time since stroke onset less than 1 week. A total of 1980 AIS patients were initially included. The exclusion criteria were: (i) lack of dysarthria assessment or laboratory data within 24 h of admission (n = 88); (ii) absence of 3-month post-discharge follow-up or inability to assess the 90-day modified Rankin Scale (mRS) score (*n* = 145); and (iii) missing lymphocyte count, albumin, or having abnormal/extreme PNI values (more than three standard deviations from the mean) (*n* = 29). Ultimately, 1,697 participants were included in the final analysis. The participant selection process is illustrated in Figure 1.

### Ethical approval and consent

This study was granted ethical approval by the Ethics Review Committee of Shenzhen Second People’s Hospital (Ethics Approval Number: 2023–305-01PJ). All participants provided written informed consent prior to enrollment. Furthermore, this research was carried out in strict adherence to the principles outlined in the Declaration of Helsinki, upholding the applicable ethical standards and regulations as specified in the declaration section.

### Variables

#### Prognostic nutritional index

PNI was calculated using the following formula: PNI = Serum Albumin (g/L) + 5 × Lymphocyte Count (10^9^/L) (19). Both the serum albumin concentration and lymphocyte count were measured within 24 h of the patient’s hospital admission.

#### Assessment of clinical outcome

At 90 days post-AIS onset, centrally trained follow-up personnel conducted patient assessments through in-person or telephone interviews. Information on functional status and all-cause mortality was collected, with data on deceased patients provided by their relatives. The mRS score was employed to evaluate the patients’ functional status. The primary endpoint of this study was the 90-day neurological function outcome, dichotomized into favorable (mRS < 3) and unfavorable (mRS ≥ 3) outcomes (20). The secondary outcome was 90-day mortality.

#### Covariates

The covariates were selected based on previous research and our clinical expertise (4, 21–23). The variables used as covariates include: (i) Continuous variables: age, hemoglobin (HGB), platelets (PLT), high-density lipoprotein cholesterol (HDL-c), homocysteine (HCY), initial National Institutes of Health Stroke Scale (NIHSS) score at admission, low-density lipoprotein cholesterol (LDL-c), Neutrophil count(NEU), red blood cell distribution width (RDW), triglycerides (TG), alanine aminotransferase (ALT), body mass index (BMI), D-dimer, fibrinogen (FIB), serum creatinine (Scr), fasting plasma glucose (FPG), and total cholesterol (TC).(ii) Categorical variables: stroke etiology, coronary heart disease (CHD), diabetes mellitus (DM), hypertension, previous stroke/transient ischemic attack (TIA), sex, atrial fibrillation (AF), and smoking status.

#### Data collection and measurement

Upon hospital admission, trained research coordinators collected baseline data on demographic characteristics and medical history. This included information on prior strokes, DM, hypertension, AF, heart disease, and smoking status. BMI was calculated as weight in kilograms divided by height in square meters. Stroke severity was assessed at admission by trained neurologists using the National Institutes of Health Stroke Scale (NIHSS). Stroke subtypes were classified according to the Trial of Org 10,172 in Acute Stroke Treatment (TOAST) criteria. Blood samples were obtained within 24 h of admission and analyzed in the laboratory department of Shenzhen Second People’s Hospital. Experienced technicians conducted rigorous quality control and measurement of laboratory parameters while maintaining the confidentiality of patients’ baseline information.

#### Handling of missing data

In this study, certain covariates had missing data, with the specific number and percentage of missing data points as follows: NIHSS score (58, 3.42%), HCY (118, 6.95%), Scr (109, 6.42%), TC (40, 2.36%), TG (39, 2.30%), HDL-c (39, 2.30%), LDL-c (39, 2.30%), FIB (4, 0.23%), FPG (4, 0.23%), Neu (4, 0.23%), HGB (4, 0.23%), PLT (4, 0.23%), and RDW (4, 0.23%). Missing data can compromise the statistical validity of the target sample during the modeling process. To minimize the bias introduced by missing variables, we performed multiple imputations on the missing data (24, 25). Covariates included in the imputation model were stroke etiology, CHD, DM, hypertension, previous stroke/TIA, sex, AF, smoking status, age, HGB, PLT, HDL-c, HCY, NIHSS score at admission, LDL-c, CRP, NEU, RDW, TG, ALT, BMI, D-dimer, FIB, Scr, FPG, and TC. The imputation was performed using a linear regression method with 10 iterations. The missing data analysis was conducted under the assumption of missing at random (MAR) (25). In addition, the data after imputation was compared with the data before imputation to assess the validity of the multiple imputation data.

### Statistical analysis

The statistical analysis was performed using R software version 3.4.3 and Empower(R) software version 4.2. A two-sided *p*-value less than 0.05 was considered statistically significant. Baseline variables were categorized based on the quartiles of PNI, and the baseline characteristics for each group were compared. Continuous variables were expressed as median (interquartile range) or mean (standard deviation), while categorical variables were presented as percentages and frequencies. Differences between PNI groups were analyzed using the chi-square (χ^2^) test for categorical variables, and the analysis of variance (ANOVA) and Kruskal-Wallis H test were employed for continuous variables.

This study employed univariate and multivariate binary logistic regression to establish three distinct models to investigate the association between PNI and the risk of unfavorable outcomes and mortality at 90 days post-AIS. The models used were as follows: (i) Model I: No adjustment for covariates;(ii) Model II: Adjusted for age and sex;(iii) Model III: Adjusted for smoking, HDL-c, stroke etiology, Scr, PLT, age, LDL-c, hypertension, FPG, TG, NIHSS score, CHD, DM, sex.

To enhance the reliability of the study findings, we conducted several sensitivity analyses. First, we transformed PNI into a categorical variable based on quartiles and calculated the *p*-value for the trend to examine the results of PNI as a continuous variable and explore potential non-linearity. Secondly, given that obesity, DM, and hypertension are associated with the prognosis of AIS patients, patients with BMI ≥ 24 kg/m^2^, DM, and hypertension were separately excluded in sensitivity analyses to further explore the relationship between PNI and 90-day unfavorable outcomes and mortality in AIS patients (26–28). Third, considering that excluding participants with extreme PNI values may lead to selection bias, Model IV is a sensitivity analysis conducted on participants without excluding extreme PNI values. Additionally, we computed the E-value to assess the possible presence of unmeasured confounders affecting the relationship between PNI and 90-day unfavorable outcomes in AIS patients (29).

To further investigate the potential nonlinear relationship between PNI and both 90-day adverse outcomes and 90-day mortality in AIS patients, a logistic regression model with cubic spline functions was employed. In cases where a non-linear relationship was identified, a recursive approach was utilized to pinpoint the inflection point. Following this, distinct binary logistic regression models were constructed on either side of the inflection point. The best model that best represents the relationship between them was determined using the likelihood ratio test.

Stratified binary logistic regression models were employed to conduct subgroup analyses across various categories, including age, sex, TG, smoking status, CHD, and AF. In this analysis, continuous variables such as age and TG were classified according to clinically significant thresholds. Specifically, age was segmented into four categories: under 60 years, 60 to 70 years, 70 to 80 years, and 80 years or older. TG was divided into two groups based on a threshold of 1.7 mmol/L. Adjustments were made for smoking, HDL-c, stroke etiology, Scr, PLT, age, LDL-c, hypertension, FPG, TG, NIHSS score, CHD, DM, sex, while excluding the factors utilized for stratification. Likelihood ratio tests were performed to assess the presence of interaction terms by comparing models that included these terms with those that did not.

## Results

**Figure 2:**
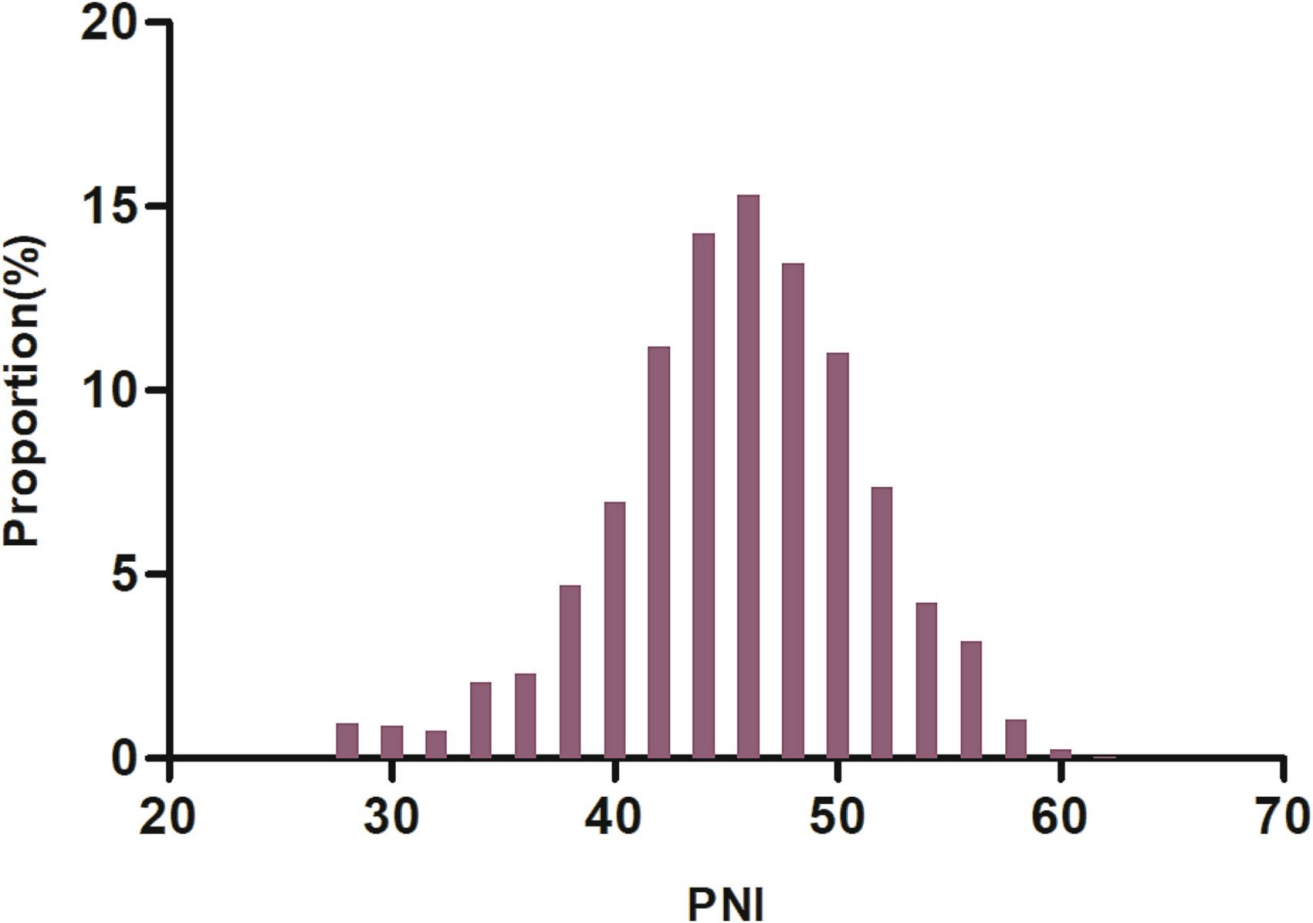
Distribution of PNI. It was approximately normally distributed, ranging from 27.4 to 61.64, with a mean ± standard deviation (SD) of 45.55 ± 5.67.

To assess the validity of the multiple imputation data, the data before multiple imputation was compared with the data after multiple imputation. It was found that there were no significant differences in baseline characteristics between the two groups, with standard deviations all less than 10% and *p*-values for inter-group comparisons greater than 0.05. This indicates that there are no significant differences between the imputed data and the original data (Supplementary Table S1). Table 1 summarized the demographic and clinical characteristics of the participants in this study. The subjects were categorized into distinct subgroups according to the quartiles of PNI (<42.10, 42.10–45.88, 45.80–49.41, ≥49.41). In comparison to the first quartile group (PNI <42.10), individuals in the higher quartile groups exhibited elevated levels of HGB, HDL-c, PLT, TC, ALB, TG, and LDL-c upon admission, while levels of Neu, Lyc, FIB, cystatin C, age, and D-dimer were found to be lower. Additionally, compared to participants in the first quartile, those in the fourth quartile had higher proportions of DM, smoking, and hypertension, while the proportions of AF and CHD were lower. Figure 2 showed the distribution of PNI, which follows a normal distribution (normality test *p*-value greater than 0.05, see Supplementary Table S2), ranging from 27.4 to 61.64, with a mean (± standard deviation, SD) of 45.55 ± 5.67.

**Table 1:** The baseline characteristics of participants.

PNI quartiles	Q1(≤42.10)	Q2(42.10–45.89)	Q3(45.89–49.45)	Q4(≥49.45)	P-value
Participants	423	423	425	426	
Age (years, mean ± SD)	73.74 ± 11.09	69.18 ± 9.47	65.35 ± 10.14	62.34 ± 11.07	<0.001
Neu (10^9^/L, mean ± SD)	6.61 ± 3.94	5.42 ± 2.71	4.99 ± 2.06	5.30 ± 2.18	<0.001
Lyc (10^9^/L, mean ± SD)	1.08 ± 0.46	1.44 ± 0.46	1.76 ± 0.46	2.25 ± 0.61	<0.001
BMI (kg/m^2^, mean ± SD)	23.50 ± 3.08	23.59 ± 3.24	23.51 ± 3.40	23.56 ± 3.32	0.973
NIHSS score (median, quartile)	5.41(2.02–7.09)	5.89(2.31–5.88)	6.16 (2.54–7.83)	6.09(2.58–7.48)	0.201
HGB (g/L, mean ± SD)	128.91 ± 23.05	137.80 ± 19.70	142.20 ± 17.43	146.46 ± 16.73	<0.001
RDW (%, mean ± SD)	40.99 ± 12.48	38.86 ± 19.49	37.12 ± 12.15	37.04 ± 11.72	<0.001
PLT (10^9^/L, mean ± SD)	199.83 ± 96.48	210.75 ± 64.11	224.35 ± 61.82	240.08 ± 65.45	<0.001
FIB (g/L, mean ± SD)	3.73 ± 1.65	3.18 ± 1.18	3.13 ± 1.32	2.91 ± 1.14	<0.001
HCY (umol/L, mean ± SD)	18.16 ± 14.99	17.93 ± 16.80	19.53 ± 37.49	16.92 ± 15.38	0.432
ALB(g/L, mean ± SD)	32.85 ± 3.95	36.88 ± 2.37	38.62 ± 2.34	41.19 ± 2.95	<0.001
FPG(mmol/L, mean ± SD)	6.75 ± 3.40	7.12 ± 3.50	6.72 ± 3.05	6.75 ± 2.78	0.207
TC (mmol/L, mean ± SD)	4.40 ± 4.69	4.53 ± 1.09	4.64 ± 1.42	4.84 ± 1.19	0.090
TG (mmol/L, mean ± SD)	1.13 (0.85–1.51)	1.30 (0.95–1.84)	1.48 (1.07–2.10)	1.65 (1.22–2.39)	<0.001
HDL-c(mmol/L, mean ± SD)	1.25 ± 2.21	1.50 ± 4.72	1.39 ± 4.76	1.47 ± 2.89	0.767
LDL-c(mmol/L, mean ± SD)	2.69 ± 1.04	2.97 ± 0.89	3.00 ± 0.89	3.21 ± 0.99	<0.001
D-dimer(mg/dL, mean ± SD)	1.06 (0.78–6.49)	0.98 (0.56–3.04)	0.72 (0.48–1.90)	0.62 (0.46–1.36)	<0.001
Scr(umol/L, median quartile)	76.91 (65.42–93.26)	77.79 (64.53–95.47)	77.91 (64.32–91.94)	78.68 (63.87–97.24)	0.801
Sex(*n*, %)					0.889
Male	265 (62.65%)	257 (60.76%)	269 (63.29%)	266 (62.44%)	
Female	158 (37.35%)	166 (39.24%)	156 (36.71%)	160 (37.56%)	
Previous stroke/TIA (*n*, %)	44(10.40%)	40 (9.46%)	39 (9.18%)	30 (7.04%)	0.502
Stroke etiology (*n*, %)					0.375
SVO	138 (32.62%)	145 (34.28%)	146 (34.35%)	129 (30.28%)	
CE	98 (23.17%)	85 (20.09%)	83 (19.53%)	76 (17.84%)	
LAA	155 (36.64%)	163 (38.53%)	156 (36.71%)	186 (43.66%)	
Undetermined	32 (7.57%)	30 (7.09%)	40 (9.41%)	35 (8.22%)	
CHD (*n*, %)	136 (32.15%)	104 (24.59%)	74 (17.41%)	63 (14.79%)	<0.001
DM	110 (26.00%)	128 (30.26%)	132 (31.06%)	132 (30.99%)	0.316
Hypertension (*n*, %)	119 (28.13%)	74 (17.49%)	42 (9.88%)	37 (8.69%)	0.001
AF (*n*, %)	79 (18.68%)	53 (12.53%)	23 (5.41%)	32 (7.51%)	<0.001
Smoking (*n*, %)	46 (10.87%)	71 (16.78%)	105 (24.71%)	120 (28.17%)	<0.001

Values are mean ± standard deviation or median (interquartile) or number (%). TC, total cholesterol; PNÍ, prognostic nutritional index; TG, triglyceride; FIB, fibrinogen; HGB, hemoglobin concentration; AST, aspartate aminotransferase; NEU, neutrophil count; RDW, red blood cell distribution width;LDL-c, low-density lipoproteins cholesterol; PLT, platelets; HCY, homocysteine; DM, diabetes mellitus; HDL-c, high-density lipoprotein cholesterol; Scr, serum creatinine; BMI, body mass index; ALB, serum albumin; AF, Atrial fibrillation; Lyc, Lymphocyte count; CHD, coronary heart disease; LAA, large artery atherosclerosis; TIA, transient ischemia attack. NIHSS, National Institute of Health stroke scale; CE, cardio embolism; SVO, small vessel occlusion.

### Incidence of unfavorable outcomes and mortality 90-day after acute ischemic stroke

**Figure 3:**
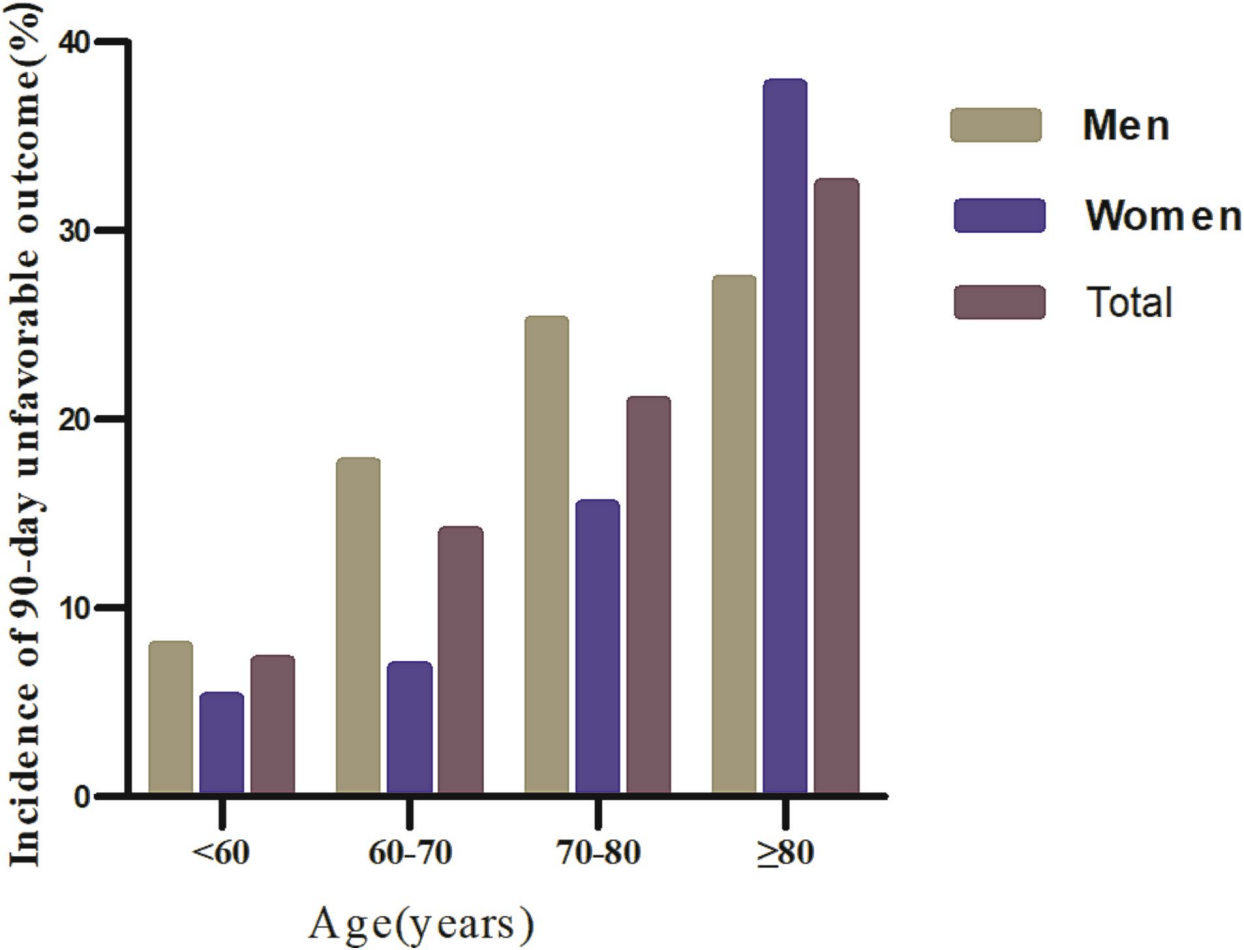
Comparative charts displayed the incidence of 90-day unfavorable outcomes across age groups, stratified by decade.

The 90-day incidence of unfavorable outcomes and mortality among AIS patients is shown in Table 2. The results indicate that 295 participants experienced unfavorable outcomes, with an overall incidence rate of 17.38%. Specifically, as shown in Figure 3, the incidence rates of unfavorable outcomes for the first to fourth quartiles of PNI were 28.13, 19.62, 9.65, and 12.21%, respectively. Additionally, 37 patients experienced mortality within 90 days after AIS, resulting in a 90-day mortality rate of 2.18%. The mortality for the first to fourth quartiles of PNI were 5.2, 2.84, 0.71, and 0%, respectively.

**Table 2:** Incidence rate of unfavorable outcome and mortality 90-day after acute ischemic stroke (%).

	Participants	Unfavorable outcome events (N)	Incidence of unfavorable (%)	Death events (N)	Mortality (%)
Total	1,697	295	17.38(15.58, 19.19)	37	2.18(1.47, 2.88)
Q1	423	119	28.13(23.83,32.43)	22	5.2(3.08,7.34)
Q2	423	83	19.62(15.82, 23.42)	12	4.8(1.25,4.42)
Q3	425	41	9.65(6.83, 12.47)	3	0.71(0.01,1.51)
Q4	426	52	12.21(9.09,15.33)	0	-
P for trend			<0.001		

N, Number; mRS, modified Rankin scale.

Regardless of age group, age stratification based on age < 60 years, 50–70 years, 70–80 years, and ≥ 80 years resulted in a higher incidence of 90-day unfavorable outcomes in men with AIS. In addition, the incidence of 90-day unfavorable outcomes increased with age in both men and women (Figure 3).

### Results of univariate analysis using binary logistic regression model

The results of the univariate analysis using a binary logistic regression model indicated significant associations between certain factors and 90-day unfavorable outcomes in AIS. Specifically, the risk of adverse outcomes was positively correlated with BMI, Neu, RDW, FPG, DD, and age. Conversely, Lyc, PLT, ALB, PNI, and TG showed a negative relationship with unfavorable outcomes. Additionally, patients with hypertension, DM, CHD, a history of stroke/TIA, and AF had a higher risk of adverse outcomes (all *p* < 0.05). Similarly, Neu, RDW, FPG, DD, and age were positively correlated with 90-day mortality, while Lyc, PLT, ALB, PNI, and TG were negatively correlated with 90-day mortality. The 90-day mortality rate was higher in patients with DM, CHD, and AF (Supplementary Table S3).

### Relationship between PNI and 90-day adverse outcomes and mortality in AIS patients

To further examine the association between the PNI and the risk of unfavorable outcomes at 90 days in patients with AIS, three binary logistic regression models were constructed (Table 3). In Model I, it was found that for each one-unit increase in PNI, the incidence of 90-day unfavorable outcomes decreased by 8.4% (OR = 0.916, 95% CI: 0.896, 0.937). In Model II, after adjusting solely for demographic factors, a one-unit increase in PNI was associated with a 5.6% reduction in the incidence of 90-day unfavorable outcomes among AIS patients (OR = 0.944, 95% CI: 0.921, 0.967). In Model III, it was observed that for every one-unit increase in PNI, the incidence of 90-day unfavorable outcomes in AIS patients decreased by 4.9% (OR = 0.951, 95% CI: 0.925, 0.979).

**Table 3:** Association of PNI with 90-day unfavorable outcomes and 90-day mortality follow acute ischemic stroke in different models.

Exposure	Model I (OR,95%CI) p	Model II(OR,95%CI) p	Model III(OR,95%CI) p
90-day mortality			
PNI(per 1-unit)	0.863 (0.820, 0.909) <0.001	0.876 (0.829, 0.926) <0.001	0.868 (0.806, 0.934) <0.001
90-day unfavorable outcome			
PNI(per 1-unit)	0.916 (0.896, 0.937) <0.001	0.944 (0.921, 0.967) <0.001	0.951 (0.925, 0.979) <0.001
PNI quartiles			
Q1	Ref	Ref	Ref
Q2	0.624 (0.453, 0.859) 0.004	0.787 (0.564, 1.097) 0.157	0.832 (0.579, 1.195) 0.320
Q3	0.273 (0.186, 0.401) <0.001	0.394 (0.264, 0.590) <0.001	0.525 (0.374, 0.660) <0.001
Q4	0.355 (0.248, 0.509) <0.001	0.595 (0.403, 0.877) 0.009	0.676 (0.437, 1.045) 0.078
P for trend	<0.001	<0.001	0.008

Model I: No covariates were adjusted. Model II: Age and sex were adjusted. Model III: Age, BMI, sex, LDL-c, TG, HDL-c, Scr, FPG, hypertension, PLT, CHD, smoking, DM, stroke etiology, and initial NIHSS score were adjusted.

Besides, PNI, initially treated as a continuous variable, was converted into a categorical variable and subsequently reintroduced into the model in this form. The multivariable regression analysis revealed that, when using participants in the first quartile as a reference group, those in the second quartile exhibited an OR of 0.832 (95% CI: 0.579, 1.195), while individuals in the third quartile had an OR of 0.525 (95% CI: 0.374, 0.660), and those in the fourth quartile showed an OR of 0.676 (95% CI: 0.437, 1.045). The distribution of confidence intervals indicated that there was no statistically significant difference in the risk of an unfavorable 90-day outcome for AIS participants in the second and third quartiles of the PNI compared to participants in the first quartile. Conversely, participants in the third quartile had a significantly reduced risk of 90-day unfavorable outcome. Additionally, the test for the trend in effect size was statistically significant (P for trend <0.05) (Table 3, Model III).

Moreover, regarding the association between PNI and 90-day mortality in patients with AIS, the OR along with their 95%CI in Models I, II, and III were found to be 0.863 (0.820, 0.909), 0.876 (0.829, 0.926), and 0.868 (0.806, 0.934), respectively. These results suggest that for each one-unit increase in PNI, the risk of 90-day mortality for AIS patients decreased by 13.7, 12.4, and 13.2% in Models I, II, and III, respectively (Table 3).

### Sensitivity analysis

To ensure the robustness of the study results, a series of sensitivity analyses were conducted (Table 4). First, participants with a BMI ≥ 24 kg/m^2^ were excluded. After adjusting for confounding variables, it was observed that PNI was negatively related to the risk of 90-day unfavorable outcomes in AIS patients (OR = 0.930, 95% CI: 0.895, 0.966). The OR (95% CI) for the relationship between PNI and 90-day mortality in AIS patients was 0.868 (0.783, 0.961). Secondly, excluding participants with DM resulted in similar findings, with ORs (95% CI) for 90-day adverse outcomes and 90-day mortality of 0.958 (0.925, 0.993) and 0.835 (0.763, 0.914), respectively. Furthermore, even when restricting the participants to non-hypertensive patients, a negative association between PNI and unfavorable outcomes in AIS patients could still be observed (OR = 0.959, 95% CI: 0.921, 0.999). The OR (95% CI) for the relationship between PNI and 90-day mortality was 0.907 (0.801, 0.996). Another sensitivity analysis was performed on participants (n = 1,842) without excluding extreme values of PNI. The results found a negative correlation between PNI and 90-day adverse outcomes in AIS patients (OR = 0.947, 95% CI: 0.920, 0.974). The OR value (95% CI) for PNI and 90-day mortality was 0.892 (0.824, 0.964). Additionally, the E-value was calculated to assess the potential impact of unmeasured confounding factors on the study results. The E-value obtained was 1.94, which exceeds the relative risk of unmeasured confounding factors and PNI (1.57), indicating that the influence of unmeasured or unknown confounding factors on the relationship between PNI and 90-day unfavorable outcomes in AIS patients is minimal. The results of all sensitivity analyses confirm the reliability of the study findings.

**Table 4:** Association of PNI with 90-day unfavorable outcomes and 90-day mortality follow acute ischemic stroke in different sensitivity analyses.

Exposure	90-day poor outcome	90-day mortality
	OR (95%CI) p-value	OR (95%CI) p-value
Model I	0.930 (0.895, 0.966) <0.001	0.868 (0.783, 0.961) 0.007
Model II	0.958 (0.925, 0.993) 0.017	0.835 (0.763, 0.914) <0.001
Model III	0.959 (0.921, 0.999) 0.045	0.907 (0.801, 0.996) 0.048
Model IV	0.947 (0.920, 0.974) <0.001	0.892 (0.824, 0.964) 0.004

Model I was sensitivity analysis in participants without BMI ≥ 24 kg/m^2^ (*n* = 978). Adjusted age, sex, LDL-c, TG, HDL-c, Scr, FPG, hypertension, PLT, CHD, smoking, DM, Stroke etiology, and initial NHISS score were adjusted. Model II was sensitivity analysis in participants without DM (*n* = 1,195). Adjusted age, BMI, sex, LDL-c, TG, HDL-c, Scr, FPG, hypertension, PLT, CHD, smoking, Stroke etiology, and initial NHISS score. Model III was sensitivity analysis in participants without hypertension (*n* = 738). Adjusted age, BMI, sex, LDL-c, TG, HDL-c, Scr, FPG, PLT, CHD, smoking, DM, Stroke etiology, and initial NHISS score. Model IV is a sensitivity analysis for participants (*n* = 1842) without excluding extreme values of PNI. Adjusted age, BMI, sex, LDL-c, TG, HDL-c, Scr, FPG, hypertension, PLT, CHD, smoking, DM, stroke etiology, and initial NIHSS score. OR odds ratios; CI, confidence; Ref: reference.

### Generalized additive model (GAM) for addressing nonlinear relationship between PNI and 90-day adverse outcomes and mortality

**Figure 4:**
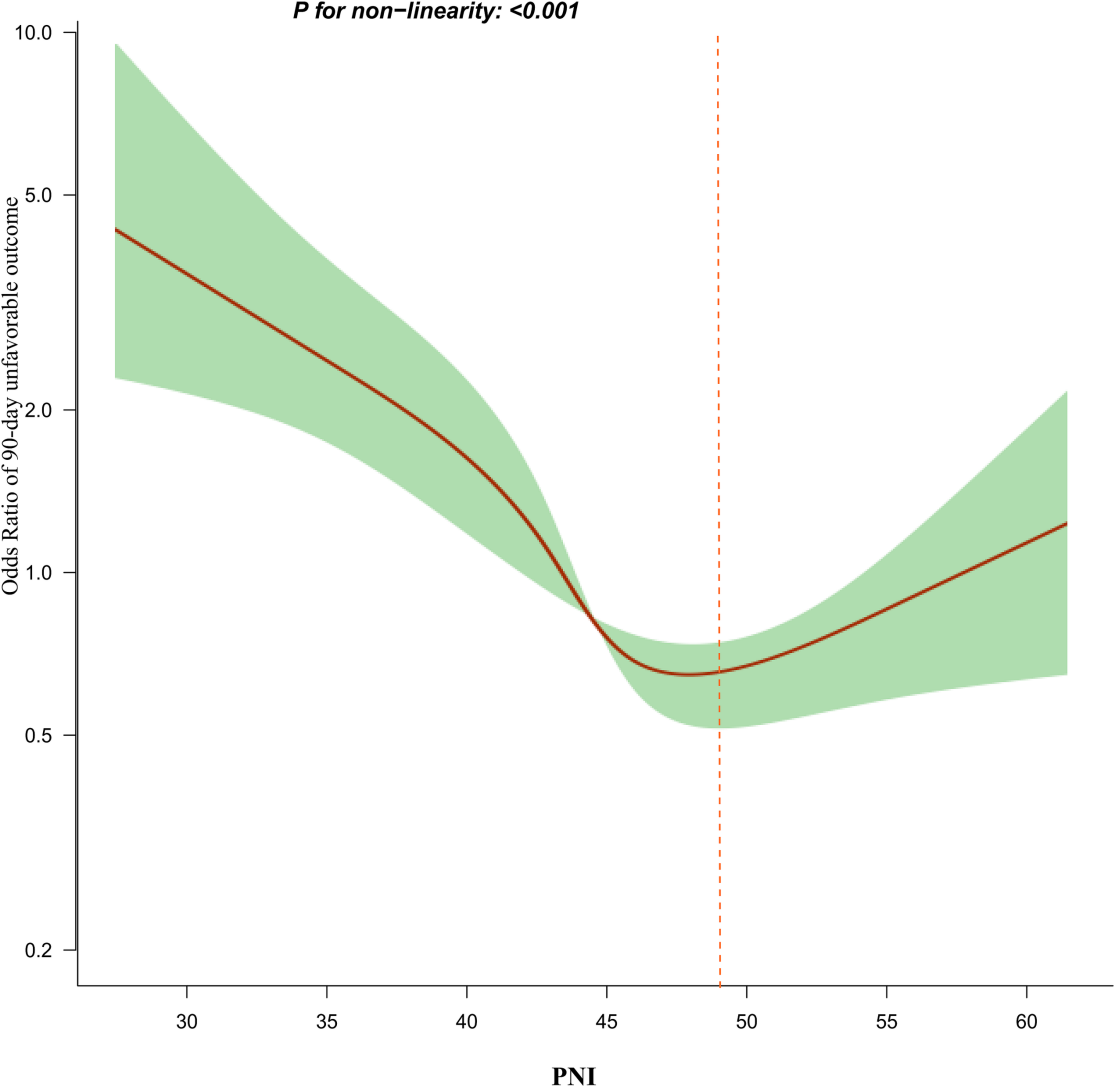
The nonlinear relationship between PNI and the risk of 90-day unfavorable outcomes.

Utilizing a logistic regression model with cubic spline function, a non-linear association between PNI and 90-day unfavorable outcomes in patients with AIS was identified (p for nonlinearity <0.05, Figure 4). The covariates adjusted in this analysis included age, BMI, sex, TG, HDL-c, HGB, FPG, hypertension, PLT, CHD, smoking, DM, stroke etiology, and initial NHISS score. A recursive method revealed an inflection point for PNI at 49.3. Following this, a piecewise logistic regression model was employed to estimate the OR and CI on either side of the inflection point. To the left of this point, the OR reflecting the relationship between PNI and the risk of 90-day unfavorable outcomes was 0.910 (95% CI: 0.880, 0.942). In contrast, to the right of the inflection point, the OR was 1.149 (95% CI: 0.998, 1.249), but this finding did not reach statistical significance (Table 5). In addition, further analysis using a logistic regression model that included cubic spline functions found that the nonlinear relationship between PNI and 90-day mortality in AIS patients was not established (p for nonlinearity >0.05).

**Table 5:** Relationship between PNI and 90-day unfavorable outcomes analyzed by two-piecewise linear regression model.

Outcome: 90-day unfavorable outcome	OR (95%CI) *p*-value
Fitting model by two-piecewise linear regression	
Inflection point of PNI	49.3
PNI < 49.3 (per 1-unit)	0.910 (0.880, 0.942) <0.001
PNI ≥ 49.3 (per 1-unit)	1.149 (0.998, 1.249) 0.103
P for log-likelihood ratio test	<0.001

Adjusted age, BMI, sex, LDL-c, TG, HDL-c, Scr, FPG, hypertension, PLT, CHD, smoking, DM, Stroke etiology, and initial NHISS score. OR,odds ratios; CI, confidence; Ref: reference.

### Subgroup analysis results

In all predefined or exploratory subgroup analyses (Table 6), there were no significant interactions between PNI and age, sex, smoking status, AF, TG, and CHD (all *p* ≥ 0.05). This indicates that these factors do not alter or modify the relationship between PNI and 90-day unfavorable outcomes or 90-day mortality in AIS patients.

**Table 6:** Stratified associations of PNI with 90-day unfavorable outcomes and 90-day mortality follow AIS in different sensitivity analyses. by age, sex, CHD, Previous stroke/TIA, smoking status, and AF.

Characteristic		90-day poor outcome	P for interacion	90-day mortality	P for interacion
		OR (95%CI) *p* value		OR (95%CI) P value	
Age(years)			0.113		0.667
<60	402	0.998 (0.896, 1.112) 0.967		0.929 (0.802, 1.075) 0.323	
60–70	562	0.930 (0.884, 0.979) 0.005		0.838 (0.743, 0.945) 0.004	
70–80	444	0.965 (0.922, 1.011) 0.137		0.889 (0.751, 1.053) 0.173	
≥80	289	0.919 (0.873, 0.967) 0.001		0.844 (0.745, 0.957) 0.008	
Sex			0.495		0.127
Male	1,057	0.945 (0.913, 0.978) 0.001		0.910 (0.838, 0.988) 0.024	
Female	640	0.962 (0.922, 1.004) 0.073		0.737 (0.627, 0.866) <0.001	
TG			0.130		0.340
<1.7 mmol/L	1,133	0.921 (0.891, 0.952) <0.001		0.879 (0.812, 0.952) 0.001	
≥1.7 mmol/L	564	0.961 (0.824, 1.120) 0.607		0.819 (0.716, 0.937) 0.004	
Previous stroke/TIA			0.765		0.999
<6.1	1,264	0.949 (0.922, 0.976) <0.001		0.869 (0.809, 0.934) <0.001	
≥6.1	560	0.980 (0.795, 1.208) 0.849		-	
CHD			0.490		0.813
≤200	1,320	0.946 (0.916, 0.977) <0.001		0.873 (0.799, 0.953) 0.002	
>200	377	0.963 (0.920, 1.008) 0.110		0.859 (0.770, 0.959) 0.007	
AF			0.442		0.452
No	1,510	0.855 (0.788, 0.927) <0.001		0.855 (0.788, 0.927) <0.001	
Yes	187	0.925 (0.864, 0.991) 0.027		0.901 (0.799, 1.016) 0.090	
Smoking			0.161		0.357
No	1,355	0.941 (0.912, 0.969) <0.001		0.859 (0.796, 0.926) <0.001	
Yes	342	0.988 (0.926, 1.054) 0.717		0.955 (0.758, 1.203) 0.696	

Above model adjusted for age, sex, LDL-c, TG, HDL-c, Scr, FPG, hypertension, PLT, CHD, smoking, DM, Stroke etiology, and initial NHISS score. In each case, the Model is not adjusted for the stratification variable. OR, odds ratios; CI, confidence.

## Discussion

This study found an independent negative correlation between PNI and 90-day adverse outcomes, as well as between PNI and 90-day mortality in AIS patients. Additionally, a saturation effect curve was observed, with a PNI inflection point at 49.3. Different relationships between PNI and 90-day unfavorable outcomes were observed on either side of this inflection point.

The PNI was initially recognized as a reliable predictor of postoperative complications and is now considered a valuable nutritional marker for various cancers, including breast, gastric, and esophageal cancers (30–33). Furthermore, several studies have indicated that PNI, as a nutritional marker, correlates with the clinical outcomes of cardiovascular diseases, such as heart failure and coronary atherosclerotic heart disease (34–36). Based on this evidence, we hypothesize that PNI may have a negative correlation with the prognosis of patients experiencing AIS. However, research on the association between PNI and the prognosis of AIS patients remains limited and inconsistent. A study conducted in China revealed that among AIS patients undergoing intravenous thrombolysis, those with a lower PNI (≤ 44.5) exhibited a 1.25-fold increase in the likelihood of poor prognosis (mRS ≥ 3) at 3 months compared to those with a higher PNI (> 44.5) (adjusted OR = 2.250, CI: 1.192–4.249) (17). Similarly, research from Turkey involving 158 AIS patients found that low PNI values were linked to elevated in-hospital mortality rates, extended hospital stays, and a higher risk of infection among individuals with AIS (37). Another investigation involving 171 AIS patients indicated that those with a PNI < 38 experienced a 2.793-fold increase in the incidence of poor prognosis at 6 months compared to patients with a PNI ≥ 38 (OR = 3.793, 95% CI: 1.117–12.882). However, when PNI was analyzed as a continuous variable in relation to six-month adverse outcomes, no significant association was identified (OR = 0.973, 95% CI: 0.904–1.047, *p* = 0.468) (38). The discrepancies in these findings may stem from several factors, including variations in study populations and sample sizes, as well as differences in the covariates adjusted for in each study. Additionally, the potential influence of nonlinear associations should be considered. Our research supports the hypothesis that an increase in PNI correlates with a decrease in both the incidence of 90-day unfavorable outcomes and 90-day mortality in AIS patients. Notably, this study examined PNI as both a categorical and continuous variable, thereby enhancing the understanding of its relationship with 90-day unfavorable outcomes and mortality while minimizing information loss. Sensitivity analyses conducted on participants with BMI < 24 kg/m^2^, without hypertension or DM, further validated the relationship among these individuals, confirming the robustness of our results. In conclusion, clarifying the relationship between PNI and the prognosis of AIS patients offers a novel perspective for improving the rehabilitation and management of stroke patients, ultimately enhancing their health status and quality of life. Additionally, this may encourage clinicians to reevaluate risk assessment and strategies for improving stroke prognosis.

The precise mechanism underlying the negative association between the PNI and short-term outcomes in patients with AIS remains inadequately elucidated. This relationship may be linked to both nutritional and immune status. PNI integrates serum albumin levels and total lymphocyte counts, where serum albumin serves as a marker for the body’s protein reserves; low serum albumin levels are frequently indicative of inadequate nutritional status and chronic illnesses (39–41). Meanwhile, total lymphocyte count provides insight into immune system functionality, with diminished levels potentially signaling immune deficiency (42, 43). Together, these two parameters are utilized to evaluate a patient’s overall nutritional and inflammatory condition. Adequate nutritional status is known to facilitate recovery, lower the risk of infections, and enhance the overall prognosis for stroke patients. In contrast, malnutrition can adversely affect these processes, heightening the likelihood of complications and prolonging recovery time (44, 45).

Besides, after stratifying participants based on PNI quartiles, the results from the multivariate adjustment model showed that, compared to the first quartile of PNI, the OR for the second, third, and fourth quartiles were 0.832, 0.525, and 0.676, respectively. This indicates that the incidence of 90-day unfavorable outcomes in the fourth PNI quartile might be slightly higher than in the third quartile. In other words, from the first to the third PNI quartile, AIS patients showed an overall declining trend in adverse outcome risk, which stopped and reversed in the fourth quartile. This essentially suggests a potential non-linear relationship between PNI and adverse outcomes, with a possible inflection point in the third or fourth PNI quartile. To verify our hypothesis, we used logistic regression with cubic splines. This analysis revealed a nonlinear relationship between PNI and 90-day unfavorable outcomes in AIS patients, with the inflection point for PNI being 49.3. The inflection point located at the end of the third quartile is consistent with our previous hypothesis. Moreover, linear regression analysis of the overall trend as a continuous variable indicates that higher PNI values are associated with a lower risk of adverse outcomes. Although the risk of adverse outcomes in the fourth PNI quartile appears slightly higher than in the third quartile, the risk of adverse outcomes in the fourth quartile remains lower than in the first and second quartiles. Consequently, the fitted linear relationship for the entire population still demonstrates an overall declining trend, which is also comprehensible. Furthermore, a non-linear relationship represents a connection between two variables where changes in one variable do not correspond to constant changes in the other. Relationships between non-linear entities can still be predicted but are more complex than linear relationships. Therefore, considering the intricacy of their relationship, a non-linear relationship may more closely approximate the true connection between PNI and the risk of unfavorable outcomes in AIS patients. Further analysis using a logistic regression model with cubic spline functions found that the nonlinear relationship between PNI and 90-day mortality in AIS patients was not established. This may be due to the fact that there were no patients who died in the fourth quartile of PNI.

The two-piecewise linear regression analysis found that for PNI values below the inflection point, each unit increase in PNI results in a 9% reduction in the risk of 90-day adverse outcomes. However, when PNI exceeds 49.3, there is no statistically significant difference in their relationship. In other words, the incidence of unfavorable outcomes in AIS patients decreases with higher PNI values, but once PNI exceeds 49.3, further increases do not lead to a further decrease in the incidence of 90-day unfavorable outcomes. Further analysis revealed that participants with PNI ≥ 49.3 had higher levels of TG, TC, LDL-c, PLT, and NIHSS scores compared to those with PNI < 49.3. Additionally, the proportion of AIS patients with PNI ≥ 49.3 who smoked, had hypertension, and had CHD was higher (Supplementary Table S4). However, these indicators are closely related to adverse outcomes in AIS (46–50). In the population with PNI less than 49.3, the levels of these risk factors were lower, resulting in a weaker impact on adverse outcomes for AIS patients, thus making the effect of PNI relatively stronger. Conversely, when PNI exceeds 49.3, the presence of these risk factors enhances their negative impact on adverse outcomes for AIS patients, thereby weakening the effect of PNI on adverse outcomes. This may explain the nonlinear relationship between PNI and 90-day unfavorable outcomes in AIS patients. This finding aids in clinical consultation and provides a basis for decision-making in optimizing stroke rehabilitation. Clinicians can develop more personalized treatment plans based on the patient’s PNI value. For patients with AIS who have a PNI value of less than 49.3, more aggressive therapeutic measures, such as enhanced nutrition, more frequent monitoring, intensified secondary prevention strategies, and more aggressive rehabilitation strategies, may be needed to reduce the risk of adverse outcomes.

This study presents several notable advantages. First, it investigates the association between PNI and unfavorable outcomes in patients with AIS by treating PNI as both a continuous and categorical variable (based on quartiles). This dual approach minimizes the loss of information and effectively quantifies the relationship between PNI and patient prognosis. Second, in contrast to earlier research, this study significantly improves the analysis of nonlinear relationships. Third, it utilizes multiple imputation techniques to manage missing data, thereby enhancing statistical power and mitigating potential biases arising from absent covariate information. Furthermore, to bolster the reliability of the findings, several sensitivity analyses were performed. These analyses included transforming independent variables, calculating E-values to evaluate the potential impact of unmeasured confounding variables, and re-examining the relationship between PNI and short-term outcomes in AIS patients after excluding individuals with BMI ≥ 24 kg/m^2^, as well as those with hypertension and DM.

Several potential limitations must be acknowledged. First, the study participants were exclusively Chinese, raising the question of whether these findings can be generalized to other ethnic groups, which requires additional validation. Second, this investigation evaluated PNI and other relevant parameters solely at baseline, without exploring how fluctuations in PNI over time might influence the prognosis of patients with AIS. This aspect represents a crucial area for future research, which will aim to gather more comprehensive data, including longitudinal changes in PNI. Third, like all observational studies, this research may be affected by unmeasured or uncontrolled confounding variables, even after accounting for recognized potential confounders. Nonetheless, we calculated E-values, which suggest that unmeasured or uncontrolled confounding factors are unlikely to account for our results. Fourth, in our prospective cohort study, due to a follow-up period of only 90 days, the relationship between PNI and 90-day adverse outcomes was initially focused on, while the specific timing of death in some AIS patients was overlooked. As a result, the Cox proportional hazards model or the Cox model with cubic spline functions was not used to analyze the relationship between PNI and clinical prognosis. However, considering that the follow-up time was limited to 90 days and the primary outcome variable was 90-day unfavorable outcomes, with the majority of participants (97.82%) reaching the 90-day endpoint and only a few deaths having uncertain follow-up outcomes, it is believed that survival bias is unlikely to significantly impact our results. In the future, plans will be made to increase the sample size and clarify the timing of death and follow-up duration to study their relationship more comprehensively. Fifth, the exclusion of participants with extreme PNI values may introduce selection bias; however, sensitivity analyses demonstrated that including these extreme values yielded results consistent with those obtained from analyses excluding them regarding the relationship between PNI and unfavorable outcomes in AIS patients. Sixth, attrition bias may arise from excluding follow-up participants. Nonetheless, a comparison of baseline characteristics between those who completed follow-up and those who did not revealed no significant differences in nearly all characteristics (Supplementary Table S5). Finally, it is essential to highlight that this study is observational in nature, indicating only an independent association between PNI and short-term prognosis in AIS patients without establishing a causal link between the two.

## Conclusion

This study reveals an independent negative relationship between PNI and both 90-day unfavorable outcomes and 90-day mortality in patients with AIS. Additionally, a nonlinear relationship between PNI and 90-day unfavorable outcomes was observed. Specifically, when the PNI value is below 49.3, a clear inverse relationship exists between the two. These findings provide further insights for optimizing rehabilitation strategies and clinical management, as well as clinical consultation for AIS patients.

## Data Availability

The raw data supporting the conclusions of this article will be made available by the authors, without undue reservation.

## Glossary

HDL-c: high-density lipoprotein cholesterol
FIB: fibrinogen
TIA: transient ischemia attack
Scr: serum creatinine
NEU: neutrophil count
ALB: serum albumin
CHD: coronary heart disease
RDW: red blood cell distribution width
TC: total cholesterol
SVO: small vessel occlusion
HCY: homocysteine
Lyc: lymphocyte count
DM: diabetes mellitus
PLT: platelets
TG: triglyceride
NIHSS: National Institute of Health stroke scale
LAA: large artery atherosclerosis
BMI: body mass index
HGB: hemoglobin concentration
CE: cardio embolism
LDL-c: low-density lipoproteins cholesterol
