# Supplementary material for: Nonlinear dose–response relationship between prognostic nutritional index and short-term outcome in acute ischemic stroke: a prospective cohort study
Source: Front Nutr. 2025 Mar 10;12:1529146. doi: 10.3389/fnut.2025.1529146 (PMC11930808; doi:10.3389/fnut.2025.1529146)
Supplement: Supplementary file 1 [file Table_1.docx]

**Nonlinear dose-response relationship between prognostic nutritional index and short-term outcome in acute ischemic stroke: a prospective cohort study.**

**Running title:** PNI and outcome of AIS

Juan Wang^1#^, Xiongbin Cao^2#^, Shan zeng^3^, Li Zhou^3^, Jianping Huang^3^, Yong Han^3*^, Zhe Deng^3*^

^1^Department of Emergency, Shenzhen Yantian District People's Hospital, Southern University of Science and Technology Yantian Hospital, Shenzhen, 518081, Guangdong, China.

^2^Neurology Department, Shenzhen Longhua District Central Hospital, Shenzhen 518110, Guangdong, China.

^3^Department of Emergency, Shenzhen Second People's Hospital, The First Affiliated Hospital of Shenzhen University, Shenzhen 518035, Guangdong Province, China.

Juan Wang and Xiongbin Cao have contributed equally to this work.

*Corresponding author

Yong Han

Department of Emergency, Shenzhen Second People's Hospital

No.3002 Sungang Road, Futian District,

Shenzhen 518035,

Guangdong Province,

China.

Hanyong511023@163.com

*Corresponding author

Zhe Deng

Department of Emergency, Shenzhen Second People's Hospital

No.3002 Sungang Road, Futian District,

Shenzhen 518035,

Guangdong Province,

China.

Dengz163@163.com

**Table S1 Comparison of baseline characteristics before and after multiple imputations.**

|  | **Before multiple imputation** | **After multiple imputation** | Standardize diff. | P-value | P-value* |
| --- | --- | --- | --- | --- | --- |
| N | 1697 | 1697 |  |  |  |
| Age (years, mean ±SD) | 67.64 ± 11.30 | 67.64 ± 11.30 | 0.00 (-0.07, 0.07) | 1.000 | 1.000 |
| Neu (10^9^/L, mean ±SD) | 5.58 ± 2.89 | 5.58 ± 2.89 | 0.00 (-0.07, 0.07) | 0.980 | 0.971 |
| Lyc (10^9^/L, mean ±SD) | 1.64 ± 0.66 | 1.63 ± 0.66 | 0.00 (-0.07, 0.07) | 0.950 | 0.953 |
| BMI (kg/m^2^, mean ±SD) | 23.54 ± 3.26 | 23.54 ± 3.26 | 0.00 (-0.07, 0.07) | 1.000 | 1.000 |
| NIHSS score (median, quartile) | 5.89 ± 5.57 | 5.89 ± 5.60 | 0.00 (-0.07, 0.07) | 0.986 | 0.708 |
| HGB (g/L, mean ±SD) | 138.88 ± 20.44 | 138.86 ± 20.42 | 0.00 (-0.07, 0.07) | 0.977 | 0.968 |
| RDW (%, mean ±SD) | 38.49 ± 14.40 | 38.50 ± 14.40 | 0.00 (-0.07, 0.07) | 0.980 | 0.968 |
| PLT (10^9^/L, mean ±SD) | 218.87 ± 74.80 | 218.80 ± 74.80 | 0.00 (-0.07, 0.07) | 0.978 | 0.981 |
| FIB (g/L , mean ±SD) | 3.24 ± 1.37 | 3.24 ± 1.37 | 0.00 (-0.07, 0.07) | 0.995 | 0.999 |
| HCY (umol/L, mean ±SD) | 18.49 ± 23.19 | 18.13 ± 23.19 | 0.02 (-0.05, 0.08) | 0.658 | 0.879 |
| ALB(g/L , mean ±SD) | 37.39 ± 4.25 | 37.39 ± 4.25 | 0.00 (-0.07, 0.07) | 1.000 | 1.000 |
| FPG(mmol/L , mean ±SD) | 6.84 ± 3.18 | 6.83 ± 3.19 | 0.00 (-0.07, 0.07) | 0.966 | 0.973 |
| TC (mmol/L , mean ±SD) | 4.59 ± 2.55 | 4.60 ± 2.58 | 0.00 (-0.06, 0.07) | 0.911 | 0.972 |
| TG (mmol/L, mean ±SD) | 1.68 ± 1.30 | 1.68 ± 1.30 | 0.00 (-0.07, 0.07) | 0.993 | 0.934 |
| HDL-c(mmol/L, mean ±SD) | 1.43 ± 3.81 | 1.40 ± 3.81 | 0.01 (-0.06, 0.07) | 0.860 | 0.884 |
| LDL-c(mmol/L, mean ±SD) | 2.98 ± 0.97 | 2.97 ± 0.97 | 0.01 (-0.06, 0.07) | 0.854 | 0.864 |
| D-dimer(mg/dL, mean ±SD) | 4.31 ± 13.49 | 4.31 ± 13.49 | 0.00 (-0.07, 0.07) | 1.000 | 1.000 |
| Sex(n,%) |  |  | 0.00 (-0.07, 0.07) | 1.000 | - |
| Male | 1057 (62.29%) | 1057 (62.29%) |  |  |  |
| Female | 640 (37.71%) | 640 (37.71%) |  |  |  |
| Previous stroke/TIA(n, %) | 141 (8.31%) | 141 (8.31%) | 0.00 (-0.07, 0.07) | 1.000 |  |
| DM | 502 (29.58%) | 502 (29.58%) | 0.00 (-0.07, 0.07) | 1.000 |  |
| CHD (n, %) | 377 (22.22%) | 377 (22.22%) | 0.00 (-0.07, 0.07) | 1.000 |  |
| Stroke etiology(n, %) |  |  | 0.00 (-0.07, 0.07) | 1.000 |  |
| SVO | 558 (32.88%) | 558 (32.88%) |  |  |  |
| CE | 342 (20.15%) | 342 (20.15%) |  |  |  |
| LAA | 660 (38.89%) | 660 (38.89%) |  |  |  |
| Undetermined | 137 (8.07%) | 137 (8.07%) |  |  |  |
| Hypertension | 959 (56.51%) | 959 (56.51%) | 0.00 (-0.07, 0.07) | 1.000 |  |
| Smoking (n, %) | 342 (20.15%) | 342 (20.15%) | 0.00 (-0.07, 0.07) | 1.000 |  |
| AF | 187 (11.02%) | 187 (11.02%) | 0.00 (-0.07, 0.07) | 1.000 |  |

Values are mean ± standard deviation or median (interquartile) or number (%)

TC, total cholesterol; PNÍ, prognostic nutritional index; TG, triglyceride; FIB, fibrinogen; HGB, hemoglobin concentration; AST, aspartate aminotransferase; NEU, neutrophil count; RDW, red blood cell distribution width ;LDL-c, low-density lipoproteins cholesterol; PLT, platelets; HCY, homocysteine; HDL-c, high-density lipoprotein cholesterol; Scr, serum creatinine; BMI, body mass index; ALB, serum albumin; AF, Atrial fibrillation; Lyc, Lymphocyte count; CHD, coronary heart disease; LAA, large artery atherosclerosis; TIA, transient ischemia attack. NIHSS, National Institute of Health stroke scale; CE, cardio embolism; SVO, small vessel occlusion;。

**Table S2 Normality Test of PNI**

| **Normality test** | | | | | | | |
| --- | --- | --- | --- | --- | --- | --- | --- |
|  | Kolmogorov-Smirnov test | | |  | Shapiro-Wilk test | | |
|  | **statistics** | Number | p-value |  | **statistics** | Number | p-value |
| PNI | 0.134 | 1697 | .085 |  | 0.989 | 1697 | .073 |

Table S3 Results of Univariate Analysis Using Binary Logistic Regression Model

|  | Statistics | Unfavorable (OR.95%CI) p | Mortality (OR.95%CI)p |
| --- | --- | --- | --- |
| Neu (10^9^/L, mean ±SD) | 5.578 ± 2.886 | 1.316 (1.260, 1.374) <0.00001 | 1.217 (1.133, 1.307) <0.00001 |
| Lyc (10^9^/L, mean ±SD) | 1.634 ± 0.662 | 0.324 (0.257, 0.409) <0.00001 | 0.034 (0.014, 0.084) <0.00001 |
| BMI (kg/m^2^, mean ±SD) | 23.539 ± 3.260 | 1.051 (1.012, 1.092) 0.00983 | 1.069 (0.971, 1.176) 0.17535 |
| NIHSS score (median, quartile) | 5.889 ± 5.598 | 0.986 (0.963, 1.009) 0.22589 | 0.983 (0.924, 1.046) 0.58338 |
| HGB (g/L, mean ±SD) | 138.861 ± 20.425 | 1.006 (0.999, 1.012) 0.07550 | 0.992 (0.977, 1.008) 0.33525 |
| RDW (%, mean ±SD) | 38.500 ± 14.395 | 1.010 (1.001, 1.019) 0.03644 | 1.014 (1.002, 1.026) 0.02305 |
| PLT (10^9^/L, mean ±SD) | 218.796 ± 74.798 | 0.997 (0.995, 0.999) 0.00219 | 0.994 (0.989, 0.999) 0.01822 |
| HCY (umol/L, mean ±SD) | 18.134 ± 23.186 | 1.004 (0.999, 1.010) 0.09972 | 1.001 (0.989, 1.013) 0.88644 |
| FIB (g/L , mean ±SD) | 3.238 ± 1.371 | 1.312 (1.206, 1.428) <0.00001 | 1.058 (0.844, 1.328) 0.62337 |
| ALB(g/L , mean ±SD) | 37.393 ± 4.250 | 0.956 (0.929, 0.984) 0.00224 | 0.948 (0.883, 1.017) 0.13889 |
| PNI | 45.551 ± 5.674 | 0.916 (0.896, 0.937) <0.00001 | 0.863 (0.820, 0.909) <0.00001 |
| FPG(mmol/L , mean ±SD) | 6.832 ± 3.192 | 1.072 (1.036, 1.110) 0.00007 | 1.138 (1.069, 1.213) 0.00006 |
| TC (mmol/L , mean ±SD) | 4.601 ± 2.579 | 1.006 (0.963, 1.050) 0.80000 | 0.806 (0.600, 1.082) 0.15128 |
| TG (mmol/L, mean ±SD) | 1.680 ± 1.301 | 0.821 (0.716, 0.943) 0.00509 | 0.481 (0.280, 0.827) 0.00808 |
| HDL-c(mmol/L, mean ±SD) | 1.404 ± 3.814 | 0.976 (0.917, 1.039) 0.45033 | 0.993 (0.892, 1.106) 0.90382 |
| LDL-c(mmol/L, mean ±SD) | 2.969 ± 0.971 | 1.039 (0.914, 1.180) 0.56210 | 0.736 (0.507, 1.067) 0.10542 |
| Sex(n,%) |  |  |  |
| Male | 1057 (62.286%) | Ref | Ref |
| Female | 640 (37.714%) | 0.864 (0.665, 1.124) 0.27552 | 0.606 (0.291, 1.259) 0.17938 |
| Age (years, mean ±SD) | 67.640 ± 11.295 | 1.057 (1.044, 1.070) <0.00001 | 1.049 (1.017, 1.082) 0.00221 |
| D-dimer(mg/dL, mean ±SD) | 4.308 ± 13.493 | 1.042 (1.030, 1.054) <0.00001 | 1.016 (1.005, 1.027) 0.00465 |
| Previous stroke/TIA(n, %) |  |  |  |
| No | 1556 (91.691%) | 1.0 | - |
| Yes | 141 (8.309%) | 0.127 (0.047, 0.346) 0.00005 | - |
| DM(n, %) |  |  |  |
| No | 1195 (70.418%) | Ref | Ref |
| Yes | 502 (29.582%) | 1.076 (0.819, 1.413) 0.60027 | 0.761 (0.356, 1.624) 0.47994 |
| Hypertension (n, %) |  |  |  |
| No | 738 (43.489%) | Ref | Ref |
| Yes | 959 (56.511%) | 0.698 (0.543, 0.898) 0.00517 | 0.517 (0.266, 1.004) 0.05140 |
| CHD (n, %) |  |  |  |
| No | 1320 (77.784%) | Ref | Ref |
| Yes | 377 (22.216%) | 2.780 (2.123, 3.641) <0.00001 | 3.433 (1.783, 6.611) 0.00022 |
| AF (n, %) |  |  |  |
| No | 1510 (88.981%) | Ref | Ref |
| Yes | 187 (11.019%) | 2.718 (1.945, 3.800) <0.00001 | 5.899 (3.003, 11.585) <0.00001 |
| Smoking |  |  |  |
| No | 1355 (79.847%) | Ref | Ref |
| Yes | 342 (20.153%) | 0.571 (0.400, 0.816) 0.00211 | 0.344 (0.105, 1.126) 0.07779 |
| Stroke etiology (n, %) |  |  |  |
| SVO | 58 (32.882%) | Ref | Ref |
| CE | 342 (20.153%) | 1.236 (0.881, 1.735) 0.21959 | 0.762 (0.325, 1.786) 0.53194 |
| LAA | 660 (38.892%) | 0.770 (0.566, 1.048) 0.09620 | 0.490 (0.222, 1.078) 0.07619 |
| Undetermined | 137 (8.073%) | 1.300 (0.821, 2.058) 0.26320 | 0.471 (0.108, 2.065) 0.31846 |

TC, total cholesterol; PNÍ, prognostic nutritional index; TG, triglyceride; FIB, fibrinogen; HGB, hemoglobin concentration; AST, aspartate aminotransferase; NEU, neutrophil count; RDW, red blood cell distribution width ;LDL-c, low-density lipoproteins cholesterol; PLT, platelets; HCY, homocysteine; HDL-c, high-density lipoprotein cholesterol; Scr, serum creatinine; BMI, body mass index; ALB, serum albumin; AF, Atrial fibrillation; Lyc, Lymphocyte count; CHD, coronary heart disease; LAA, large artery atherosclerosis; TIA, transient ischemia attack. NIHSS, National Institute of Health stroke scale; CE, cardio embolism; SVO, small vessel occlusion.

Table S4 Comparison of Baseline Characteristics of Participants with PNI <49.3 and PNI ≥49.3

|  | <49.3 | >=49.3 | P-value |
| --- | --- | --- | --- |
| N | 1264 | 433 |  |
| Neu (10^9^/L, mean ±SD) | 5.68 ± 3.09 | 5.28 ± 2.17 | 0.012 |
| BMI (kg/m^2^, mean ±SD) | 23.53 ± 3.24 | 23.56 ± 3.32 | 0.852 |
| Lyc (10^9^/L, mean ±SD) | 1.42 ± 0.54 | 2.25 ± 0.61 | <0.001 |
| NIHSS score (median, quartile) | 4.00 (2.00-8.00) | 4.10 (2.01-7.98) | 0.503 |
| HGB (g/L, mean ±SD) | 136.28 ± 20.94 | 146.41 ± 16.69 | <0.001 |
| RDW (%, mean ±SD) | 39.02 ± 15.17 | 36.99 ± 11.75 | 0.012 |
| PLT (10^9^/L, mean ±SD) | 211.58 ± 76.54 | 239.85 ± 65.12 | <0.001 |
| HCY (umol/L, mean ±SD) | 18.57 ± 25.32 | 16.85 ± 15.31 | 0.182 |
| FIB (g/L , mean ±SD) | 3.35 ± 1.43 | 2.91 ± 1.13 | <0.001 |
| ALB(g/L) | 36.10 ± 3.84 | 41.16 ± 2.95 | <0.001 |
| FPG (mmol/L , mean ±SD) | 6.86 ± 3.32 | 6.75 ± 2.78 | 0.540 |
| TC (mmol/L, mean ±SD) | 4.52 ± 2.90 | 4.83 ± 1.19 | 0.031 |
| TG (mmol/L, mean ±SD) | 1.57 ± 1.28 | 1.99 ± 1.32 | <0.001 |
| HDL-c(mmol/L, mean ±SD) | 1.38 ± 4.09 | 1.47 ± 2.86 | 0.683 |
| LDL-c(mmol/L, mean ±SD) | 2.89 ± 0.95 | 3.21 ± 0.99 | <0.001 |
| Age (years, mean ±SD) | 69.45 ± 10.82 | 62.36 ± 11.02 | <0.001 |
| D-dimer (mg/dL, mean ±SD) | 5.21 ± 15.40 | 1.67 ± 3.40 | <0.001 |
| Sex(n,%) |  |  | 0.881 |
| Male | 786 (62.18%) | 271 (62.59%) |  |
| Female | 478 (37.82%) | 162 (37.41%) |  |
| Previous stroke/TIA (n, %) | 111 (8.78%) | 30 (6.93%) | 0.228 |
| DM | 368 (29.11%) | 134 (30.95%) | 0.471 |
| Hypertension (n, %) | 698 (55.22%) | 261 (60.28%) | 0.067 |
| CHD (n, %) | 313 (24.76%) | 64 (14.78%) | <0.001 |
| AF (n, %) | 154 (12.18%) | 33 (7.62%) | 0.009 |
| smoking (n, %) | 221 (17.48%) | 121 (27.94%) | <0.001 |
| Stroke etiology(n, %) |  |  | 0.169 |
| SVO | 424 (33.54%) | 134 (30.95%) |  |
| CE | 265 (20.97%) | 77 (17.78%) |  |
| LAA | 473 (37.42%) | 187 (43.19%) |  |
| Undetermined | 102 (8.07%) | 35 (8.08%) |  |

Values are mean ± standard deviation or median (interquartile) or number (%)

TC, total cholesterol; PNÍ, prognostic nutritional index; TG, triglyceride; FIB, fibrinogen; HGB, hemoglobin concentration; AST, aspartate aminotransferase; NEU, neutrophil count; RDW, red blood cell distribution width ;LDL-c, low-density lipoproteins cholesterol; PLT, platelets; HCY, homocysteine; HDL-c, high-density lipoprotein cholesterol; Scr, serum creatinine; BMI, body mass index; ALB, serum albumin; AF, Atrial fibrillation; Lyc, Lymphocyte count; CHD, coronary heart disease; LAA, large artery atherosclerosis; TIA, transient ischemia attack. NIHSS, National Institute of Health stroke scale; CE, cardio embolism; SVO, small vessel occlusion;。

Table S5 Baseline Characteristics of Participants Who Completed Follow-Up and Those Who Did Not

|  | Completed follow-up visits | No follow-up visits | P-value |
| --- | --- | --- | --- |
| N | 1697 | 145 |  |
| Age (years, mean ±SD) | 67.64 ± 11.30 | 66.46 ± 11.59 | 0.229 |
| Neu (10^9^/L, mean ±SD) | 5.58 ± 2.89 | 5.92 ± 3.49 | 0.174 |
| Lyc (10^9^/L, mean ±SD) | 1.63 ± 0.66 | 1.60 ± 0.64 | 0.512 |
| BMI (kg/m^2^, mean ±SD) | 23.54 ± 3.26 | 23.50 ± 3.37 | 0.892 |
| NIHSS score (median, quartile) | 4.00 (2.00-8.00) | 4.00 (2.00-7.00) | 0.173 |
| HGB (g/L, mean ±SD) | 138.86 ± 20.42 | 139.39 ± 20.61 | 0.764 |
| RDW (%, mean ±SD) | 38.50 ± 14.40 | 39.37 ± 12.36 | 0.481 |
| PLT (10^9^/L, mean ±SD) | 218.80 ± 74.80 | 222.88 ± 80.52 | 0.531 |
| FIB (g/L , mean ±SD) | 3.24 ± 1.37 | 3.31 ± 1.50 | 0.549 |
| HCY (umol/L, mean ±SD) | 13.70 (10.12-19.30) | 13.85 (9.93-20.10) | 0.637 |
| ALB(g/L, mean ±SD) | 37.39 ± 4.25 | 37.23 ± 4.27 | 0.653 |
| FPG(mmol/L , mean ±SD) | 6.83 ± 3.19 | 6.93 ± 3.43 | 0.715 |
| TC (mmol/L , mean ±SD) | 4.60 ± 2.58 | 4.49 ± 1.13 | 0.613 |
| TG (mmol/L, mean ±SD) | 1.68 ± 1.30 | 1.88 ± 1.83 | 0.088 |
| HDL-c(mmol/L, mean ±SD) | 1.40 ± 3.81 | 1.33 ± 2.95 | 0.811 |
| LDL-c(mmol/L, mean ±SD) | 2.97 ± 0.97 | 2.90 ± 0.89 | 0.409 |
| D-dimer (mg/dL, mean ±SD) | 4.31 ± 13.49 | 3.07 ± 6.97 | 0.276 |
| Sex(n,%) |  |  | 0.349 |
| Male | 1057 (62.29%) | 96 (66.21%) |  |
| Female | 640 (37.71%) | 49 (33.79%) |  |
| Stroke etiology (n, %) |  |  | 0.089 |
| SVO | 558 (32.88%) | 38(26.20%) |  |
| CE | 342 (20.15%) | 26(17.93%) |  |
| LAA | 660 (38.89%) | 54(37.24%) |  |
| Undetermined | 137 (8.07%) | 27(18.62%) |  |
| Hypertension | 959 (56.51%) | 10 (6.90%) | 0.552 |
| Hypertension (n, %) | 959 (56.51%) | 79 (54.48%) | 0.636 |
| AF (n, %) | 187 (11.02%) | 20 (13.79%) | 0.310 |
| Smoking (n, %) | 342 (20.15%) | 33 (22.76%) | 0.455 |

Values are mean ± standard deviation or median (interquartile) or number (%)

TC, total cholesterol; PNÍ, prognostic nutritional index; TG, triglyceride; FIB, fibrinogen; HGB, hemoglobin concentration; AST, aspartate aminotransferase; NEU, neutrophil count; RDW, red blood cell distribution width ; LDL-c, low-density lipoproteins cholesterol; PLT, platelets; HCY, homocysteine; HDL-c, high-density lipoprotein cholesterol; Scr, serum creatinine; BMI, body mass index; ALB, serum albumin; AF, Atrial fibrillation; Lyc, Lymphocyte count; CHD, coronary heart disease; LAA, large artery atherosclerosis; TIA, transient ischemia attack. NIHSS, National Institute of Health stroke scale; CE, cardio embolism; SVO, small vessel occlusion.
